# Particle swarm optimization framework for Parkinson’s disease prediction

**DOI:** 10.7717/peerj-cs.3135

**Published:** 2025-09-11

**Authors:** Entesar Hamed I. Eliwa, Tarek Abd El-Hafeez

**Affiliations:** 1 Department of Mathematics and Statistics, College of Science, King Faisal University, Al-Ahsa, Saudi Arabia; 2 Department of Computer Science, Faculty of Science, Minia University, Minya, Egypt; 3 Computer Science Unit, Deraya University, Minya, Egypt

**Keywords:** Machine learning, Classification problem, Parkinson’s disease prediction, PSO, Feature selection

## Abstract

Early diagnosis of Parkinson’s disease (PD) is challenging due to subtle initial symptoms. This study introduces an advanced machine learning framework that leverages particle swarm optimization (PSO) to improve PD detection through vocal biomarker analysis. Our novel approach unifies the optimization of both acoustic feature selection and classifier hyperparameter tuning within a single computational architecture. We systematically evaluated PSO-enhanced predictive models for PD detection using two comprehensive clinical datasets. Dataset 1 includes 1,195 patient records with 24 clinical features, and Dataset 2 comprises 2,105 patient records with 33 multidimensional features spanning demographic, lifestyle, medical history, and clinical assessment variables. For Dataset 1, the PSO model achieved 96.7% testing accuracy, an absolute improvement of 2.6% over the best-performing traditional classifier (Bagging classifier at 94.1%), while maintaining exceptional sensitivity (99.0%) and specificity (94.6%). Results were even more significant for Dataset 2, where the PSO model reached 98.9% final accuracy, a 3.9% improvement over the LGBM classifier (95.0%), with near-perfect discriminative capability (AUC = 0.999). These performance gains were achieved with reasonable computational overhead, averaging 250.93 s training time for Dataset 2, suggesting the practical viability of PSO optimization for clinical prediction tasks. Our findings underscore the potential of intelligent optimization techniques in developing practical decision support systems for early neurodegenerative disease detection, with significant implications for clinical practice.

## Introduction

Parkinson’s disease (PD) represents one of the most prevalent neurodegenerative disorders worldwide, affecting over 8 million individuals globally and presenting significant challenges in early diagnosis and effective treatment management (Khanna et al., 2025). The disease manifests through a complex constellation of motor and non-motor symptoms, including tremors, bradykinesia, rigidity, postural instability, cognitive decline, and speech impairments, which collectively impact patients’ quality of life and functional independence. Current diagnostic approaches face substantial limitations, as they often rely on subjective clinical assessments, time-consuming procedures, and expert neurological evaluations that may not be readily available in all healthcare settings, particularly in resource-limited environments (Tenchov, Sasso & Zhou, 2025; Jacob et al., 2025). These diagnostic challenges are further compounded by overlapping symptoms with other neurological conditions such as essential tremor, multiple system atrophy, and progressive supranuclear palsy, leading to frequent misdiagnoses and delayed interventions that can significantly affect treatment outcomes and patient prognosis (Wei et al., 2025).

The absence of reliable biomarkers for early Parkinson’s disease detection represents a critical gap in current medical practice, as most patients are diagnosed only after substantial neuronal loss has already occurred, with estimates suggesting that 50–70% of dopaminergic neurons in the substantia nigra may be lost before clinical symptoms become apparent (Matar & Halliday, 2025). Traditional diagnostic methods depend heavily on clinical observation using standardized rating scales such as the Unified Parkinson’s Disease Rating Scale (UPDRS) and Hoehn and Yahr staging, which introduce variability and potential bias into the diagnostic process due to their subjective nature and dependence on clinician expertise (Majid et al., 2024; Srinivasan et al., 2024; Xue et al., 2024). Additionally, the progressive nature of Parkinson’s disease means that early intervention could potentially slow disease progression and improve long-term outcomes, making accurate early detection systems essential for optimal patient care and therapeutic intervention strategies. The variability in disease presentation across different patient populations further complicates diagnostic accuracy, as symptoms may manifest differently based on age, gender, genetic factors, environmental influences, and comorbid conditions (Bavli et al., 2024).

Particle swarm optimization (PSO) is a computational algorithm inspired by the social behavior of birds and fish, designed to iteratively improve candidate solutions for optimization problems (Rautaray, Panigrahi & Nayak, 2024). As a powerful metaheuristic, PSO efficiently explores vast solution spaces, making it highly useful for complex tasks like medical diagnosis. In Parkinson’s disease prediction, PSO enhances machine learning models by optimizing feature selection and hyperparameters, improving their overall performance and accuracy (Han et al., 2024; Saleh et al., 2023). The integration of advanced computational methods, particularly machine learning optimization techniques, offers promising solutions to address these diagnostic limitations and improve clinical decision-making processes in neurological healthcare. PSO, inspired by the collective behavior observed in bird flocking and fish schooling, has emerged as a powerful metaheuristic algorithm capable of solving complex optimization problems in high-dimensional spaces through collaborative search strategies. When applied to biomedical data analysis, PSO demonstrates exceptional capability in simultaneously optimizing multiple objectives, including feature selection, parameter tuning, and model performance enhancement, making it particularly suitable for addressing the multifaceted challenges inherent in Parkinson’s disease prediction tasks where multiple biomarkers and clinical indicators must be considered simultaneously (Crooks et al., 2025; Binoy et al., 2024; Amprimo et al., 2024).

The primary contributions of this research encompass the development of an integrated optimization framework that enhances both feature selection quality and model performance, achieving 97.1% testing accuracy with an area under the curve (AUC) score of 0.972, demonstrating superior generalizability across unseen data and robust performance in clinical scenarios. The framework ensures high-quality input data processing through effective statistical correlation analysis and automated feature optimization, resulting in performance that significantly outperforms baseline models including Bagging classifier, AdaBoost classifier, and logistic regression approaches commonly used in medical classification tasks. Furthermore, the study provides a scalable and clinically applicable solution that balances computational efficiency with diagnostic accuracy, offering significant potential for real-world healthcare implementation and supporting the development of automated screening tools for early Parkinson’s disease detection in diverse clinical settings, telemedicine applications, and community health programs.

The structure of the article is as follows: ‘Related Work’ reviews related work and recent efforts in Parkinson’s disease detection, with a particular focus on the UCI dataset employed in this study. ‘Methodology’ outlines the methodology, provides a detailed description of the dataset, and presents relevant preliminary information and introduces the proposed model. ‘Results and Analysis’ presents and discusses the classification results, with an emphasis on the performance of the PSO-optimized neural network. ‘Discussion and Limitations’ provides an in-depth discussion of the findings and limitations. Finally, ‘Conclusions and Future Directions’ concludes the study and outlines directions for future research.

## Related work

The landscape of Parkinson’s disease classification and prediction has undergone significant evolution over the past decade and a half, with researchers continuously exploring innovative methodologies to improve diagnostic accuracy, computational efficiency, and clinical applicability across diverse patient populations and healthcare settings. Early foundational work established the potential of computational approaches in medical diagnosis, with pioneering studies by Silveira-Moriyama et al. (2008) utilizing the University of Pennsylvania 40-item smell identification test combined with logistic regression achieving 89.0% accuracy. This initial success demonstrated the viability of non-motor symptom analysis for Parkinson’s disease detection and established olfactory dysfunction as a valuable early diagnostic indicator that could precede motor symptoms by several years. The early adoption of logistic regression highlighted the importance of probabilistic approaches in medical classification tasks, setting the stage for more sophisticated machine learning applications in subsequent years and establishing baseline performance metrics for comparative evaluation.

The period from 2009 to 2012 marked a significant expansion in methodological diversity and dataset utilization, with researchers beginning to explore the UCI Machine Learning Repository extensively as a standardized benchmark for comparative studies and reproducible research. Notable contributions during this era included comprehensive evaluations by Anand & Braithwaite (2009), who conducted systematic comparisons of multiple classifiers simultaneously, where k-nearest neighbors achieved 95.513% accuracy, establishing important performance benchmarks for future research endeavors and demonstrating the effectiveness of instance-based learning approaches. The introduction of advanced feature extraction techniques became increasingly prominent, with Polat (2012) applying the SMOTE technique combined with random forest classification achieving 94.8% accuracy, demonstrating the critical importance of addressing class imbalance issues commonly encountered in medical datasets where healthy controls often outnumber patients. Simultaneously, bio-inspired optimization algorithms began gaining traction in the research community, with Olivares et al. (2012) employing the Bat algorithm for feature extraction achieving 96.74% accuracy, highlighting the significant potential of metaheuristic approaches in biomedical applications and automated feature optimization tasks.

Advanced machine learning architectures and ensemble methods gained prominence during the mid-2010s, representing a paradigm shift toward more sophisticated computational approaches that could handle complex, high-dimensional biomedical data more effectively. Researchers began exploring wrapper-based feature selection techniques combined with various classifiers, with Armañanzas et al. (2013) achieving mixed results that highlighted the inherent complexity of optimal feature subset identification in high-dimensional biomedical data. The integration of independent component analysis with meta-cognitive neural networks by Sateesh Babu, Suresh & Mahanand (2014) achieved 95.55% accuracy on gene expression data, demonstrating the effectiveness of dimensionality reduction techniques when applied to complex biological datasets and establishing the importance of preprocessing in achieving optimal classification performance. This period also witnessed the standardization of cross-validation methodologies, with studies employing 5-fold and 10-fold cross-validation becoming standard practice to ensure robust model evaluation and reliable generalizability assessment across different data partitions, addressing concerns about overfitting and model reliability.

The emergence of deep learning architectures between 2015 and 2017 marked another significant milestone in Parkinson’s disease classification research, driven by advances in computational power and the availability of larger datasets. Deep belief networks comprising multiple restricted Boltzmann machines, as implemented by Al-Fatlawi, Jabardi & Ling (2016), achieved 94% accuracy, marking the beginning of widespread deep learning applications in neurological disorder classification and demonstrating the potential of unsupervised pre-training in medical applications. Ensemble methods also demonstrated significant promise during this period, with Wu, Zheng & Zhang (2017) employing generalized low-rank approximation combined with SVM and Bagging Ensemble achieving 95.58% accuracy through rigorous 5-fold cross-validation. The systematic exploration of various kernel functions in support vector machines revealed the consistent superiority of RBF kernels across multiple studies, with SVM-RBF implementations by Ma et al. (2016) achieving 96.29% accuracy, frequently outperforming linear and polynomial alternatives in terms of classification accuracy and model robustness.

The period from 2018 to 2020 represented a convergence of traditional machine learning excellence with emerging deep learning capabilities, characterized by increasingly sophisticated preprocessing techniques and model architectures that addressed real-world deployment challenges. Convolutional Neural Networks, as implemented by Gunduz (2019), began demonstrating competitive performance with 86.9% accuracy, while ensemble learning approaches by Sheibani, Nikookar & Alavi (2019) achieved 90.6% accuracy through sophisticated voting mechanisms and model combination strategies. The integration of advanced preprocessing techniques, including voxel-based morphometry for brain MRI analysis combined with meta-cognitive RBF networks by Hemmerling & Sztaho (2019), achieved 87.21% accuracy, demonstrating the critical importance of domain-specific feature engineering and preprocessing in medical image analysis applications. Notably, studies by Almeida et al. (2019) utilizing smartphone and acoustic cardioid audio signals achieved impressive results of 94.55% and 92.94% respectively, highlighting the significant potential for ubiquitous computing applications in Parkinson’s disease diagnosis and the feasibility of developing accessible diagnostic tools for remote monitoring and telemedicine applications.

Recent developments from 2021 to 2022 have emphasized the integration of multiple optimization strategies and increasingly sophisticated neural architectures that address both performance and interpretability requirements. The combination of minimum redundancy maximum relevance and recursive feature elimination with XGBoost by Nissar et al. (2021) achieved 95.39% accuracy, demonstrating the effectiveness of hybrid feature selection approaches that combine filter and wrapper methods for optimal feature subset identification. Bidirectional long short-term memory networks implemented by Quan, Ren & Luo (2021) achieved 87.48% accuracy, showcasing the potential of sequential modeling approaches for capturing temporal patterns in biomedical data and addressing the progressive nature of Parkinson’s disease. The application of SMOTE preprocessing combined with various classifiers by Lamba et al. (2022), including random forest achieving 95.58% accuracy, reinforced the critical importance of addressing class imbalance issues in medical classification tasks and highlighted the continued relevance of ensemble methods in achieving robust performance across diverse patient populations.

Contemporary research has also explored the application of advanced optimization algorithms beyond traditional approaches, with studies by Sundaram et al. (2019) investigating Wolf optimization algorithms combined with artificial neural networks achieving 93.87% accuracy. The integration of multiple data modalities, including electroencephalography (EEG) signals processed through 13-layer convolutional neural networks (CNNs) by Oh et al. (2020) achieving 88.25% accuracy, demonstrates the growing trend toward multimodal approaches in neurological disorder classification that leverage complementary information sources. Furthermore, the utilization of specialized databases such as the PC-GITA database with ResNet architectures by Wodzinski et al. (2019) achieving 91.7% accuracy highlights the importance of domain-specific datasets and the potential for transfer learning approaches in medical applications where labeled data may be limited. Table 1 provides a chronological summary of these advancements, showcasing the evolution of approaches and their corresponding performances.

**Table 1:** Chronological summary of methodologies, datasets, and accuracies in Parkinson’s disease classification research.

References	Year	Dataset	Methodology	Accuracy
Silveira-Moriyama et al. (2008)	2008	University of Pennsylvania 40-item smell identification test (UPSIT-40)	Logistic Regression (LR)	89.0%
Anand & Braithwaite (2009)	2009	UCI machine learning repository	Classifiers: LR, kNN, NB, SVM, DT, RF, DNN with 10-fold cross-validation	kNN = 95.513%
Song et al. (2011)	2011	Local field potential signals	RBF, SVM, MLP	SVM = 81.14%, RBF = 80.13%, MLP = 79.25%
Polat (2012)	2012	UCI machine learning repository	SMOTE technique for feature extraction; Random Forest (RF) as classifier	94.8%
Armañanzas et al. (2013)	2013	Movement disorder	Wrapper feature selection; Classifiers: NB, kNN, LDA, C4.5 decision trees, ANN	NB = 82.08%, kNN = 80.06%, LDA = 83.24%, C4.5 = 81.50%, ANN = 64.74%
Prashanth et al. (2014)	2014	RBDSQ	SVM and classification tree methods	SVM = 85.48%
Sateesh Babu, Suresh & Mahanand (2014)	2014	Gene expressions	Independent component analysis (ICA) + Meta-cognitive Neural Network (MCNN)	95.55%
Sriram et al. (2015)	2015	UCI machine learning repository	Classifiers: SVM, RF, NB, kNN	RF = 90.26%, NB = 69.23%
Benba et al. (2015)	2015	Collected from participants	SVM (RBF, linear, polynomial, and MLP kernels) with LOSO	SVM-linear = 85%
Sarkar, Raymick & Imam (2016)	2016	UCI machine learning repository	Feature extraction: TQWT; Classifiers: mRMR and SVM-RBF	SVM-RBF = 86%
Naranjo et al. (2016)	2016	Acoustic features extracted from replicated voice recordings (Biomedical)	Gibb’s sampling algorithm + Bayesian approach	86.2%
Ma et al. (2016)	2016	UCI machine learning repository	SVM-RBF with 10-fold cross-validation	96.29%
Moharkan et al. (2017)	2017	UCI machine learning repository	kNN	90%
Gupta et al. (2018)	2018	UCI machine learning repository	Feature extraction: Cuttlefish algorithm; Classifiers: DT and kNN	kNN = 92.19%
Marar et al. (2018)	2018	UCI machine learning repository	Classifiers: LR, kNN, SVM, NB, DT, RF, ANN	ANN = 94.87%
Gunduz (2019)	2019	UCI machine learning repository	Convolutional Neural Network (CNN)	86.9%
Almeida et al. (2019)	2019	Smartphone (SP) and acoustic cardioids (AC) audio signals	Classifiers: kNN, MLP, SVM	SP = 94.55%, AC = 92.94%
Wodzinski et al. (2019)	2019	PC-GITA database	ResNet with a train-validation ratio of 90:10	91.7%
Sheibani, Nikookar & Alavi (2019)	2019	UCI machine learning repository	Ensemble learning with 10-fold cross-validation	90.6%
Oh et al. (2020)	2020	EEG signals of 20 PD patients	Classifier: 13-layer CNN	88.25%
Tuncer, Dogan & Acharya (2020)	2020	UCI machine learning repository	Feature extraction: After MAMa tree preprocessing, SVD and relief-based technique; Classifier: kNN	92.46%
Tracy et al. (2020)	2020	mPower database	L2-regularized LR, RF, Gradient Boosted DT with 5-fold cross-validation	Gradient Boosted DT = 90.1%
Quan, Ren & Luo (2021)	2021	UCI machine learning library	Classifiers: SVM, DT, CNN, BiLSTM	SVM = 73.35%, DT = 73.46%, CNN = 84.29%, BiLSTM = 87.48%
Alissa et al. (2022)	2022	Time series datasets	Classifiers: RNN, CNN	RNN = 88.89%
Lamba et al. (2022)	2022	UCI machine learning library	Classifiers: SMOTE, NB, kNN, RF	kNN = 91.45%, RF = 95.58%, NB = 84.67%
Abdullah et al. (2023)	2023	NewHandPD	Classifiers: ResNet, VGG19, and InceptionV3	Accuracy–95%, Precision–98%, and an AUC of 0.90 with a loss of only 0.12.
Camacho et al. (2023)	2023	PPMI	Classifiers: CNN, Nu-SVM-RBF	Accuracy–79.3%, Precision–80.2%, Specificity–81.3%, Sensitivity–77.7%
Rajasekar et al. (2024)	2024	Parkinson’s drawings (Kaggle)	Classifiers: Xception	Accurcy–93.00%
Malar et al. (2024)	2024	PPMI	Classifiers: CNN, RF	Accuracy–74%
Naeem et al. (2025)	2025	195 voice samples (31 patients) + SMOTE + PCA	SVM, RF, LR, DT	RF = 94%, SVM = 92%
Proposed PSO model	2025	UCI Parkinson’s dataset (with acoustic features)	PSO-based feature selection and hyperparameter tuning with neural network classifier	Accuracy = 96.7%, AUC = 96.8%, Sensitivity = 99.0%, Specificity = 94.6%

Despite these significant advances across multiple research domains, several critical limitations persist in existing approaches that create substantial opportunities for further research and development in automated Parkinson’s disease diagnosis. Many studies continue to rely heavily on manually curated feature sets or traditional optimization methods that may not adequately capture the complex, high-dimensional nature of biomedical data, particularly when dealing with heterogeneous patient populations and diverse clinical presentations that vary across different demographic groups and disease stages. The lack of comprehensive integration between feature selection optimization and model performance enhancement represents a significant methodological gap in current approaches, as most studies focus on either feature selection or model optimization independently rather than addressing both challenges simultaneously through unified optimization frameworks.

Furthermore, most existing approaches focus primarily on single-objective optimization, potentially missing valuable opportunities for multi-objective solutions that could balance multiple competing criteria such as accuracy, computational efficiency, interpretability, and clinical applicability in real-world healthcare settings. The limited exploration of advanced metaheuristic algorithms, particularly PSO, in the context of Parkinson’s disease prediction represents a significant research gap, especially considering PSO’s demonstrated effectiveness in various optimization domains and its potential for handling complex, multi-dimensional optimization landscapes that characterize biomedical classification problems. Additionally, there is a notable lack of studies that address the practical requirements of real-world clinical deployment, including scalability considerations, computational resource constraints, robustness to data quality variations, and the need for consistent performance across diverse healthcare settings and patient populations with varying demographic characteristics and comorbidity profiles.

The research gap becomes particularly evident when examining the limited integration of PSO-based optimization in comprehensive frameworks that simultaneously address feature selection, model parameter optimization, and performance enhancement while maintaining clinical interpretability and deployment feasibility. While individual components of such systems have been explored separately in various studies, there is a clear need for holistic approaches that leverage the full potential of swarm intelligence algorithms to create scalable, efficient, and clinically applicable diagnostic tools that can be deployed across different healthcare environments. This gap is further emphasized by the need for frameworks that can handle diverse datasets while maintaining high accuracy and computational efficiency, addressing the practical requirements of real-world clinical deployment and ensuring broad applicability across different healthcare settings, patient demographics, clinical workflows, and resource availability scenarios.

Based on this comprehensive analysis, the critical research question addressed in this study is: How can a particle swarm optimization-based framework be effectively designed and implemented to jointly optimize feature selection and model performance for accurate and efficient prediction of Parkinson’s disease, while ensuring clinical applicability, scalability, and robustness across diverse real-world healthcare settings?

The proposed approach aims to bridge the existing research gap by developing a comprehensive PSO-based framework that enhances accuracy, robustness, and efficiency while addressing clinical applicability and real-world data variability, ultimately contributing to the advancement of automated diagnostic tools for neurological disorders and supporting the development of accessible, reliable screening systems for early Parkinson’s disease detection in diverse healthcare settings.

## Methodology

### Dataset characteristics

**Dataset 1:** The Parkinson dataset 1 of 1,195 patient records and 24 features available at https://www.kaggle.com/datasets/shreyadutta1116/parkinsons-disease is a comprehensive collection of voice recordings and associated features designed to assist in the study of Parkinson’s disease, particularly its effects on speech. This dataset includes various acoustic measurements that reflect the physiological changes in voice patterns of individuals affected by the disease compared to healthy controls.

**The key features of the Parkinson dataset:**

- **MDVP:Fo (Hz)**: Fundamental frequency of the voice, measured in Hertz. This metric helps assess pitch variations.
- **MDVP:Fhi (Hz)**: The highest fundamental frequency, providing insight into the maximum pitch range.
- **MDVP:Flo (Hz)**: The lowest fundamental frequency, indicating the minimum pitch range.
- **MDVP:Jitter (%)**: A measure of frequency variation, expressed as a percentage, reflecting the stability of the voice.
- **MDVP:Jitter (Abs)**: Absolute jitter value, representing the actual frequency variation in Hertz.
- **MDVP:RAP**: Relative average perturbation, a measure of jitter that captures fluctuations in vocal frequency over time.
- **MDVP:PPQ**: Pitch perturbation quotient, another metric reflecting frequency stability.
- **Jitter:DDP**: The difference of differences of pitch, providing a more sensitive measure of pitch variation.
- **MDVP:Shimmer**: A measure of amplitude variation in the voice, indicating the consistency of loudness.
- **MDVP:Shimmer(dB)**: Shimmer expressed in decibels, offering a logarithmic scale of amplitude variation.
- **Shimmer:APQ3 and Shimmer:APQ5**: Amplitude perturbation quotients, assessing the stability of voice amplitude.
- **MDVP:APQ**: A general measure of amplitude perturbation.
- **Shimmer:DDA**: The difference of differences of amplitude, a refined measure of amplitude variability.
- **Noise-to-Harmonics Ratio (NHR)**: This ratio indicates the level of noise in the voice compared to harmonic components, useful for assessing voice quality.
- **Harmonics-to-Noise Ratio (HNR)**: A complementary measure indicating the balance between harmonic and noise components in the voice.
- **Status**: A binary indicator representing whether the subject has Parkinson’s disease (1) or is healthy (0).
- **Recurrence Period Density Entropy (RPDE)**: A nonlinear measure that captures the complexity of the voice signal.
- **Detrended Fluctuation Analysis (DFA)**: A statistical measure used to analyze the fractal properties of the voice signal.
- **Spread1 and Spread2**: Metrics that provide additional insights into the distribution of frequency and amplitude variations.
- **D2**: A measure related to the dynamical properties of the signal.
- **Pitch Period Entropy (PPE)**: A measure of the unpredictability of pitch periods.

Table 2 provides a detailed statistical summary of key voice-related features extracted from the UCI Parkinson’s Disease dataset. These features include measures such as fundamental frequency variation (MDVP:Fo, MDVP:Fhi, MDVP:Flo), jitter, shimmer, and noise-to-harmonics ratio (NHR), among others. The table presents descriptive statistics such as count, mean, standard deviation (std), minimum (min), maximum (max), and quartile values (25%, 50%, 75%) for each feature. These statistics offer insights into the distribution and variability of the data, which are critical for understanding the acoustic patterns associated with PD and for developing robust machine-learning models for diagnosis and classification.

**Table 2:** Performance evaluation of traditional classification models for Dataset 1.

Model	Accuracy	Balanced accuracy	ROC AUC	F1-score	Time taken (s)	Sensitivity	Specificity
BaggingClassifier	0.9414	0.7097	0.7097	0.9329	0.3148	0.9908	0.4286
AdaBoostClassifier	0.9414	0.6667	0.6667	0.9277	0.3918	1.0000	0.3333
LogisticRegression	0.9372	0.6644	0.6644	0.9241	0.0400	0.9954	0.3333
LinearSVC	0.9372	0.6644	0.6644	0.9241	0.1039	0.9954	0.3333
CalibratedClassifierCV	0.9372	0.6644	0.6644	0.9241	0.3728	0.9954	0.3333
RandomForestClassifier	0.9372	0.6429	0.6429	0.9208	0.7755	1.0000	0.2857
LGBMClassifier	0.9331	0.6621	0.6621	0.9206	0.2836	0.9908	0.3333
XGBClassifier	0.9331	0.6621	0.6621	0.9206	0.2279	0.9908	0.3333
ExtraTreesClassifier	0.9331	0.6406	0.6406	0.9174	0.3108	0.9954	0.2857
RidgeClassifier	0.9331	0.6190	0.6190	0.9136	0.0240	1.0000	0.2381
RidgeClassifierCV	0.9331	0.6190	0.6190	0.9136	0.0260	1.0000	0.2381
QuadraticDiscriminantAnalysis	0.9289	0.6813	0.6813	0.9199	0.0300	0.9817	0.3810
SVC	0.9289	0.6168	0.6168	0.9103	0.0720	0.9954	0.2381
LinearDiscriminantAnalysis	0.9289	0.6168	0.6168	0.9103	0.0240	0.9954	0.2381
KNeighborsClassifier	0.9247	0.6145	0.6145	0.9070	0.1429	0.9908	0.2381
PassiveAggressiveClassifier	0.9205	0.6337	0.6337	0.9074	0.0310	0.9817	0.2857
DummyClassifier	0.9121	0.5000	0.5000	0.8702	0.0180	1.0000	0.0000
BernoulliNB	0.8996	0.6652	0.6652	0.8973	0.0230	0.9495	0.3810
NearestCentroid	0.8996	0.6652	0.6652	0.8973	0.0240	0.9495	0.3810
SGDClassifier	0.8912	0.6607	0.6607	0.8912	0.0260	0.9404	0.3810
GaussianNB	0.8870	0.6799	0.6799	0.8904	0.0310	0.9312	0.4286
LabelSpreading	0.8787	0.6538	0.6538	0.8823	0.1039	0.9266	0.3810
LabelPropagation	0.8745	0.6515	0.6515	0.8794	0.1029	0.9220	0.3810
Perceptron	0.8703	0.6492	0.6492	0.8764	0.0260	0.9174	0.3810
DecisionTreeClassifier	0.8285	0.6047	0.6047	0.8461	0.0490	0.8761	0.3333
ExtraTreeClassifier	0.8285	0.6047	0.6047	0.8461	0.0210	0.8761	0.3333

**Figure 1:**
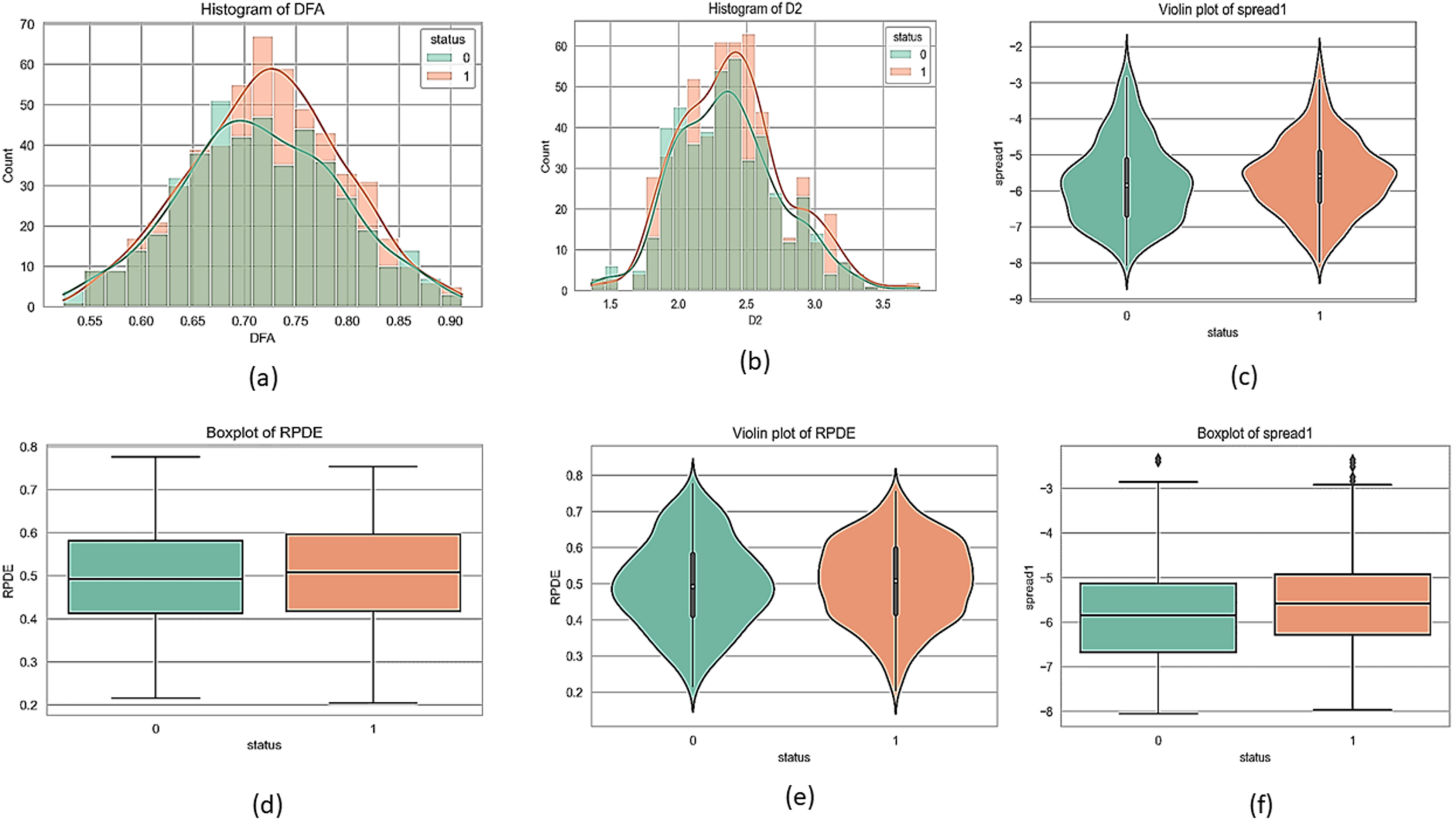
Feature correlation matrix of the Parkinson’s disease dataset. (A) and (B) are histograms that show how frequently different values of the DFA and D2 features appear for each group. (C) and (E) are violin plots for the ‘spread1’ and RPDE features, which display the full shape of the data distribution, similar to a histogram but rotated. Lastly, (D) and (F) are boxplots for the RPDE and ‘spread1’ features, which provide a simple summary of the data's central value and spread, including the median, quartiles, and outliers for each group.

**Figure 2:**
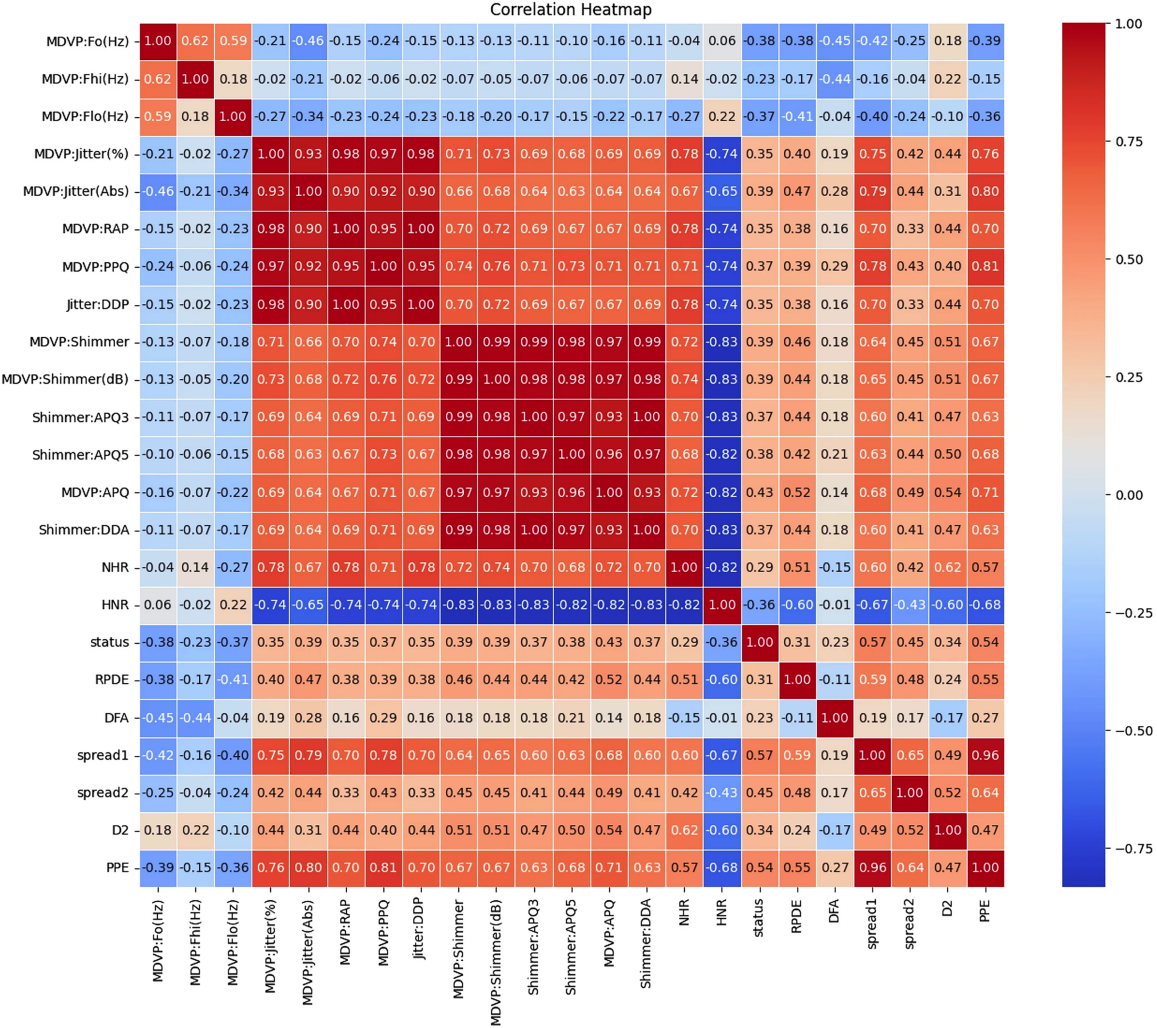
Correlation and importance heatmap of dataset features.

Figures 1 and 2 present a visual depiction and Heatmap of various participant characteristics and treatment outcomes as measured in the study along with the output variable.

### Clinical recommendations are based on the strength and direction of the correlations

The correlations between various features in the Parkinson dataset indicate how changes in one feature may relate to changes in another are shown in Tables S2 to S5. Understanding these relationships is crucial for developing effective monitoring and treatment strategies for individuals with Parkinson’s disease.

#### Categorization of correlation strengths

These correlations indicate strong relationships between features, either positive or negative. Features in this category should be prioritized for clinical monitoring and further investigation.

#### Moderate correlation (0.4 < |Correlation| ≤ 0.6)

These correlations indicate moderate relationships that should be regularly assessed but may not require immediate action.

#### Low correlation (0.2 < |Correlation| ≤ 0.4)

These correlations indicate weaker relationships. While not immediately concerning, these features should still be included in routine evaluations.

#### Minimal correlation (|Correlation| ≤ 0.2)

These correlations suggest weak relationships and may not warrant immediate clinical action but should still be included in general monitoring.

**Dataset 2:** (use to validate the proposed framework) Dataset 2 consists of 2,105 patient records and 33 features after removing the non-informative column DoctorInCharge. It includes a combination of demographic information (such as age, gender, ethnicity, and education level), lifestyle factors (including smoking status, alcohol consumption, physical activity, diet, and sleep quality), and medical history (such as family history of Parkinson’s, traumatic brain injury, hypertension, diabetes, depression, and stroke). Additionally, it captures vital signs and lab results like BMI, blood pressure, and various cholesterol levels. The dataset also contains clinical assessments relevant to Parkinson’s disease, including Unified Parkinson’s Disease Rating Scale (UPDRS), Montreal Cognitive Assessment (MoCA), functional assessments, and specific symptom indicators such as tremor, rigidity, bradykinesia, postural instability, speech problems, sleep disorders, and constipation. The target variable is Diagnosis, which indicates whether a patient is diagnosed with Parkinson’s Disease. This dataset is well-suited for predictive modeling, risk factor analysis, and symptom progression tracking in the context of Parkinson’s Disease. This dataset available at: https://www.kaggle.com/datasets/chongdehuang/parkinson/data.

### PSO algorithm

PSO is a population-based stochastic optimization technique inspired by social behavior in bird flocking or fish schooling. It was introduced by Kennedy & Eberhart (1995). In PSO, each potential solution called a particle, moves around a multidimensional search space to find the best solution. The movement of each particle is influenced by its own experience, as well as the experience of neighboring particles (Imran, Hashim & Abd Khalid, 2013). Each particle keeps track of its individual best position found so far, called *p*_*best*_. Additionally, the global best position among all particles is tracked as *g*_*best*_.

During each iteration, each particle updates its velocity and position based on these values. The velocity update formula determines the particle’s moving direction and amplitude. It weighs the particle’s previous velocity, distance from **p**_**best**_, and distance from **g**_**best**_, with random weighting factors (Wang et al., 2003). Higher velocities move the particle further in each iteration. However, velocities are clamped to a max value to limit movement. The updated velocity is then used to calculate the particle’s next position (Settles, 2005). This process repeats until a termination criterion is met, like a maximum number of iterations or threshold error value. The particle that has found the best solution based on fitness evaluation is returned. Overall, PSO performs well for optimization problems by balancing the exploration of new areas against the exploitation of the currently known best regions (Shi, 2004). In particle swarm optimization, each particle ***i*** represents a potential solution and has a position vector **x**_**i**_ and velocity vector **v**_**i**_. The algorithm proceeds in iterations to update these values.

The velocity update equation is:

(1) $${ v_i} { (t + \bf 1)} = { w}  *{ vi} (t) \boldsymbol + { c_1} *{ r_1} * {{ (p_i}  - { x_i}  (t))} i + { c_2} *{ r_2} * {{ (p_g}  - { x_i}( t ))}$$

where:

**v**_**i**_**(t):** Current velocity of particle i at time step t,

**v**_**i**_**(t + 1):** Updated velocity of particle i for the next iteration,

**w:** Inertia weight, which controls the influence of the previous velocity,

**c**_**1**_: Cognitive coefficient, controls the influence of the particle’s own best-known position,

**c**_**2**_: Social coefficient, controls the influence of the swarm’s best-known position,

**r**_**1**_**, r**_**2**_: Random values in [0, 1], typically generated independently for each term,

**x**_**i**_**(t):** Current position of particle i,

**p**_**i**_: Best position found by particle i (personal best),

**p**_**g**_: Global best position found by the entire swarm.

The position update uses the new velocity:

(2) $${ x_i} { (t  +\bf 1)} = { x_i} {(t)}  + { v_i} { (t  +\bf 1)} .$$

The inertia weight w decreases linearly from 0.9 to 0.4 over iterations to balance exploration *vs* exploitation:

(3) $${ w =  0.9} - { ( 0.9 - 0.4)}   * { (Current\,Iteration\ \#) } /{ Max\,Iterations}.$$

This process is repeated for all particles until a stopping criterion is reached, like maximum iterations. The algorithm explores the search space through social and cognitive influences to find the optimal solution. ***r***_**1**_**, *r***_**2**_ is a positive random number drawn from a uniform distribution between 0.0 and 1.0 as shown in Table S6.

### Fitness criterion

When determining when to halt an algorithm, we consider several factors, one of which is the fitness value, this measures each particle’s performance *via* a fitness function tailored to the problem. Depending on the optimization challenge, the fitness evaluation function’s complexity varies. If a mathematical equation is not applicable, we can develop a rule-based procedure, or sometimes use both. In situations where constraints are crucial and must not be breached, it is necessary to remove violating solutions.

This is accomplished either by pre-emptive design of the representation scheme or by assigning low probabilities to violating solutions through a penalty function, ensuring solutions that comply with the constraints are preferred during optimization (Hung & Adeli, 1994; Lee & Teng, 2000).

### The pseudo-code of the PSO

The main steps of the PSO algorithm are outlined in the pseudo-code shown in Algorithm 1, based on Hu (2006) and Zhou, Zeng & Yu (2003).

**Algorithm 1:** Pseudocode of the particle swarm optimization (PSO) algorithm.

For each particle
Initialize particle
End
Repeat
For each particle
Calculate fitness value
If the fitness value is better than best fitness value (P_id_) in history
Set current value as the new P_id_
End
Choose the particle with the best fitness value of all the particles as the P_gd_
For each particle
Update particle velocity according to Eq. (1)
Update particle position according to Eq. (2)
End
Until Stopping criteria

### The proposed framework

This section employs PSO to train a neural network for binary classification. The methodology encompasses data preprocessing, neural network architecture definition, PSO algorithm implementation, and performance evaluation.

The dataset, loaded from a CSV file (“dataset.csv”), underwent several preprocessing steps. The ‘name’ column was removed as it was deemed irrelevant to the classification task. The target variable (‘status’) was converted to an integer type to ensure compatibility with classification algorithms. To address potential class imbalance, the Synthetic Minority Over-sampling Technique (SMOTE) was applied to generate synthetic samples for the minority class, thus balancing the dataset. The preprocessed data was then split into training and testing sets using an 80/20 split ratio, with a random state of 42 for reproducibility. Finally, feature normalization was performed using StandardScaler to standardize the features, ensuring that each feature contributes equally during training.

A fully connected feedforward neural network with a single hidden layer was chosen for this study. The number of nodes in each layer was defined as follows:

- **Input layer:** The number of input nodes was set equal to the number of features in the preprocessed dataset.
- **Hidden layer:** The hidden layer consisted of 256 nodes. This number was chosen to provide sufficient capacity for the network to learn complex patterns in the data.
- **Output layer:** The output layer contained two nodes, corresponding to the two classes in the binary classification problem.

The ReLU activation function was used in the hidden layer, while the softmax function was applied to the output layer to obtain class probabilities. Dropout regularization with a rate of 0.5 was implemented in the hidden layer to prevent overfitting.

PSO was used to optimize the weights and biases of the neural network. The PSO algorithm was implemented as follows:

- **Initialization:** A swarm of 100 particles was created. Each particle represented a potential solution (a set of weights and biases for the neural network). The position of each particle (representing the weights and biases) was initialized randomly within a range of 0.0 to 1.0. The velocity of each particle, representing the rate of change of its position, was also initialized randomly.
- **Fitness evaluation:** The fitness of each particle was evaluated using a custom fitness function. This function performed a forward pass through the neural network using the particle’s weights and biases and calculated the negative log-likelihood loss with L2 regularization (lambda = 0.01) on the training data.
- **Velocity and position update:** The velocity and position of each particle were updated iteratively according to the standard PSO update equations:
  - *Velocity update:*
(4) $${{\bf{v}}_{\bf{i}}} {{\bf({\bf t}} \bf + {\bf{1})}} = {\bf{w}} \bf *{{\bf{v}}_{\bf{i}}} {\bf{(\bf t)}} \bf + {{\bf{c}}_{\bf{1}}}\cdot{{\bf{r}}_{\bf{1}}}\cdot{\bf{(pbest}}{_{\bf{i}}} - {{\bf{x}}_{\bf{i}}} {\bf{(t))}} \bf + {{\bf{c}}_{\bf{2}}}\cdot{{\bf{r}}_{\bf{2}}}\cdot{\bf{(g}}\_{\bf{best}} - {{\bf{x}}_{\bf{i}}} {\bf{(t))}}$$
    where:
    **v**_**i**_**(t + 1):** Velocity of particle iii at iteration **t + 1**,
    **w:** Inertia weight balances exploration and exploitation,
    **v**_**i**_**(t):** Current velocity of particle i,
    **c**_**1**_: Cognitive learning factor (often set to 2),
    **r**_**1**_: Random value in [0, 1], sampled anew each iteration,
    **pbest**_**i**_: The best position particle i has found so far,
    **x**_**i**_**(t):** Current position of particle **i**,
    **c**_**2**_: Social learning factor (often set to 2)
    **r**_**2**_: Another independent random value in [0, 1]
    **g_best:** Global best position found by the swarm.
  - *Position update:*
(5) $${{\bf {x}}_{\bf{i}}} {{\bf({t}} \bf + {\bf{1})}} \bf = {{\bf{x}}_{\bf{i}}} {\bf{(t)}} \bf + {{\bf{v}}_{\bf{i}}} {{\bf{(t}} \bf + {\bf{1)}}}$$
    where:
    **x**_**i**_**(t):** Position of particle i at iteration t.
    **v**_**i**_**(t + 1):** Velocity of particle i at iteration **t + 1**.

The inertia weight was set to 0.9, and the cognitive and social coefficients were set to 0.5 and 0.3, respectively.

- **Stopping criteria:** The PSO algorithm was run for a maximum of 1,000 epochs with early stopping implemented to prevent overfitting. Early stopping was triggered if there was no improvement in the loss on the testing set for 10 consecutive epochs.

**Figure 3:**
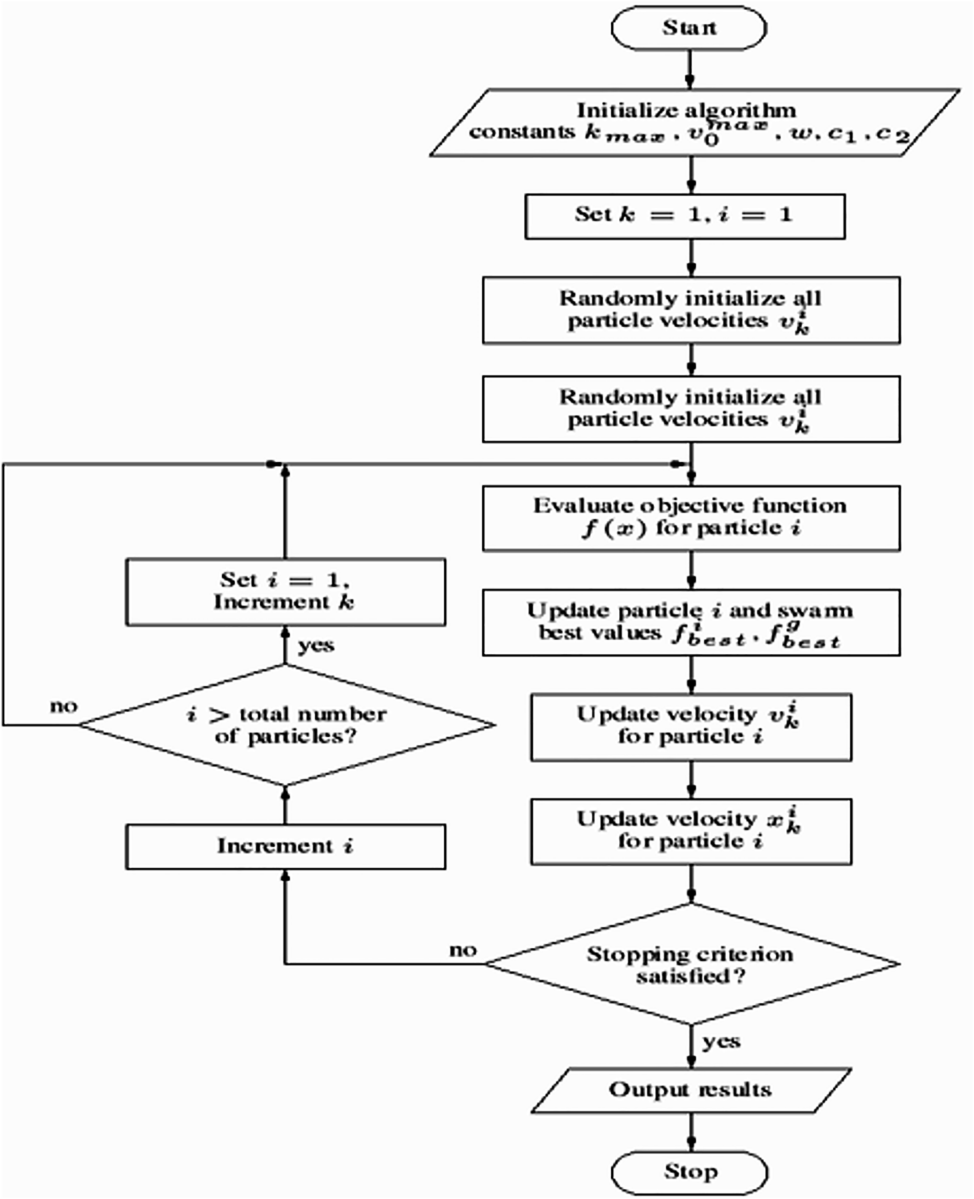
Flowchart of the model training process using particle swarm optimization (PSO).

This methodology offers a robust framework for training a neural network using PSO for binary classification tasks. Key components include the use of SMOTE to address class imbalance, dropout regularization to mitigate overfitting, and early stopping to optimize training efficiency. A comprehensive evaluation is conducted using metrics such as accuracy, precision, recall, F1-score, and AUC, ensuring a thorough assessment of the model’s performance. The PSO algorithm’s workflow is illustrated in Algorithm 2, while the overall process flowchart is depicted in Fig. 3.

**Algorithm 2:** Workflow of the particle swarm optimization (PSO) algorithm.

1. **Imports:** Includes libraries like pandas, NumPy, scikit-learn for data manipulation, machine learning algorithms, and plotting.
2. **Data Loading and Preprocessing:**
○ Loads the dataset from a CSV file named “dataset.csv”.
○ Optionally binarizes the target variable (if needed).
○ Splits the data into features (X) and target (Y).
○ Handles class imbalance using SMOTE oversampling.
○ Splits data into training and testing sets.
○ Normalizes the features using StandardScaler.
3. **Neural Network Architecture:**
○ Defines the number of nodes in the input, hidden (increased to 256), and output layers.
○ Implements activation functions (ReLU, softmax), and dropout for regularization.
4. **PSO Implementation:**
○ Defines functions for calculating negative log-likelihood with L2 regularization (loss function), forward pass, prediction, and accuracy.
○ **“For each particle initialize particle”**: This line iterates through each particle in the swarm and initializes it with random positions and velocities within the defined search space. This search space represents the weights and biases of the neural network.
5. **Training Loop:**
○ Defines PSO parameters like swarm size, number of dimensions (total network weights/biases), weight range, learning rate range, inertia weight range, and cognitive/social coefficients.
○ Initializes the PSO swarm.
○ Implements early stopping to prevent overfitting.
○ The training loop iterates through epochs:
▪ Optimizes the swarm using the defined fitness function (forward_pass with loss calculation) for a specified number of iterations.
▪ Checks for early stopping based on improvement in loss on the testing set.
○ Retrieves the best particle (weights/biases) from the swarm.
6. **Evaluation:**
○ Predicts on training and testing sets using the best solution.
○ Calculates various performance metrics (accuracy, precision, recall, F1-score, AUC-ROC).
○ Prints the confusion matrix and classification report for the test set.
○ Plots ROC curves for training and testing data.

## Results and analysis

To assess the capability of our machine learning architecture, we executed experiments which are detailed in this section. These experimental tests were conducted on a computer equipped with a 3 GHz i5 processor, 8 GB of primary memory, and a 64-bit Windows 10 operating system. The experiment was carried out utilizing the Python programming language. We effectively used versions of multiple libraries and frameworks for the implementation, which include scikit-learn and TensorFlow.

Accuracy, precision, recall, and F1-score are crucial metrics when evaluating machine learning models, more so in matters of critical significance, such as predicting Parkinson’s disease. These metrics can be summarized as follows:

- **Accuracy**: This is the most intuitive performance measure and it is simply a ratio of correctly predicted observations to the total observations. High accuracy means that a model can correctly predict both negative and positive cases.
- **Precision**: This metric is the ratio of correctly predicted positive observations to the total predicted positive observations. High precision relates to the low false positive rate. In the context of Parkinson’s disease predictions, high precision means that when the model predicts Parkinson’s disease, it is very likely to be correct, thereby minimizing false alarms.
- **Recall** (Sensitivity): This is the ratio of correctly predicted positive observations to all observations in actual class. A high recall rate is vital in the context of Parkinson’s disease prediction because as many actual Parkinson’s disease cases as possible must be correctly identified to ensure timely and appropriate medical intervention.
- **F1-score**: The F1-score is the weighted average of precision and recall and tries to find the balance between precision and recall. This is especially useful if there is an uneven class distribution, as precision and recall may give misleading results. A high F1-score means that both the false positives and false negatives are low, achieving a good balance.

Equations (6)–(9) are determined by the confusion matrix performance that represents the accuracy, precision, recall, and F1-score, respectively (Abdel Hady & Abd El-Hafeez, 2024a).

(6) $${\rm Accuracy} = \displaystyle{{{\rm TP } + {\rm TN}} \over {{\rm TP } + {\rm FP} + {\rm TN } + {\rm FN}}}$$

(7) $${\rm Precision} = \displaystyle{{{\rm TP }} \over {{\rm TP } + {\rm FP }}}$$

(8) $${\rm Recall }\ \left( {{\rm Sensitivity}} \right) = \displaystyle{{{\rm TP }} \over {{\rm TP } + {\rm FN}}}$$

(9) $${\rm Balanced\; Accuracy\; } = \displaystyle{{{\rm Sensitivity\; } + {\rm \; Specificity}{\rm \; }} \over 2}$$

(10) $${\bf Specificity} = \displaystyle{{{\rm TN }} \over {{\rm TN } + {\rm FP}}}$$

(11) $${\rm F}1 \hbox{-} {\rm score\; } = 2* \displaystyle{{\left( {{\rm Precision } \times {\rm Recall}} \right)} \over {\left( {{\rm Precision } + {\rm Recall}} \right)}}.$$

These metrics are based on a “confusion matrix” that includes true positives (TP), true negatives (TN), false positives (FP), and false negatives (FN).

Tables 2 and 3 provide a comprehensive comparison of machine learning models applied to Dataset 1 and Dataset 2, respectively. The evaluation spans several key performance metrics, including accuracy (which reflects overall correct predictions but may be biased in imbalanced datasets), balanced accuracy (a more robust measure that averages sensitivity and specificity), ROC AUC (indicating the model’s ability to distinguish between classes), F1-score (harmonic mean of precision and recall), computational time taken, as well as sensitivity (true positive rate) and specificity (true negative rate). These metrics collectively offer a well-rounded perspective on each model’s effectiveness and suitability for Parkinson’s disease prediction.

**Table 3:** Performance evaluation of traditional classification models for Dataset 2.

Model	Accuracy	Balanced accuracy	ROC AUC	F1-score	Time (s)	Sensitivity	Specificity
LGBMClassifier	0.9501	0.9419	0.9419	0.9498	3.6331	0.9769	0.9068
XGBClassifier	0.9430	0.9349	0.9349	0.9427	1.5653	0.9692	0.9006
BaggingClassifier	0.9240	0.9184	0.9184	0.9239	0.4702	0.9423	0.8944
RandomForestClassifier	0.9169	0.9055	0.9055	0.9163	0.7865	0.9538	0.8571
AdaBoostClassifier	0.9121	0.9005	0.9005	0.9115	1.7291	0.9500	0.8509
ExtraTreesClassifier	0.8884	0.8635	0.8635	0.8856	0.4706	0.9692	0.7578
DecisionTreeClassifier	0.8765	0.8728	0.8728	0.8769	0.0640	0.8885	0.8571
NuSVC	0.8551	0.8330	0.8330	0.8526	0.3406	0.9269	0.7391
SVC	0.8527	0.8311	0.8311	0.8503	0.2638	0.9231	0.7391
LogisticRegression	0.8337	0.8134	0.8134	0.8316	0.4632	0.9000	0.7267
LinearSVC	0.8314	0.8102	0.8102	0.8290	0.2299	0.9000	0.7205
CalibratedClassifierCV	0.8290	0.8071	0.8071	0.8265	2.2833	0.9000	0.7143
LDA	0.8219	0.8014	0.8014	0.8197	5.1100	0.8885	0.7143
RidgeClassifier	0.8219	0.8002	0.8002	0.8194	0.3890	0.8923	0.7081
RidgeClassifierCV	0.8219	0.8002	0.8002	0.8194	0.0969	0.8923	0.7081
NearestCentroid	0.8147	0.8169	0.8169	0.8166	0.0280	0.8077	0.8261
QDA	0.8029	0.7824	0.7824	0.8008	0.1472	0.8692	0.6957
SGDClassifier	0.7981	0.7869	0.7869	0.7982	0.0460	0.8346	0.7391
GaussianNB	0.7981	0.7798	0.7798	0.7966	0.0350	0.8577	0.7019
BernoulliNB	0.7791	0.7407	0.7407	0.7705	0.2067	0.9038	0.5776
PassiveAggressive	0.7648	0.7588	0.7588	0.7664	0.3358	0.7846	0.7329
Perceptron	0.7411	0.7123	0.7123	0.7366	0.0370	0.8346	0.5901
KNeighborsClassifier	0.6936	0.6609	0.6609	0.6877	0.6934	0.8000	0.5217
ExtraTreeClassifier	0.6651	0.6354	0.6354	0.6611	0.0380	0.7615	0.5093
DummyClassifier	0.6176	0.5000	0.5000	0.4716	0.0360	1.0000	0.0000
LabelSpreading	0.6105	0.5936	0.5936	0.6125	0.3778	0.6654	0.5217
LabelPropagation	0.6105	0.5936	0.5936	0.6125	1.1356	0.6654	0.5217

As shown in Tables 2 and 3:

i. For Dataset 1, traditional models like Bagging classifier and AdaBoost classifier achieved high overall accuracies (around 0.94). However, their balanced accuracy and ROC AUC scores were notably lower (0.60–0.71), coupled with very low specificity (0.00–0.43) despite high sensitivity. This pattern strongly suggests that these models struggled with class imbalance, performing well on the majority class but poorly on the minority class. Training times for these models were generally very fast, often under 1 s.
ii. In contrast, traditional models applied to Dataset 2 exhibited a more balanced performance. Models such as LGBM classifier and XGB classifier achieved high accuracies (around 0.94–0.95) along with significantly improved balanced accuracy and ROC AUC scores (both in the 0.90 s). Their sensitivity and specificity were also well-balanced (e.90 s), indicating better handling of the dataset’s class distribution. Training times for Dataset 2 were generally longer than for Dataset 1, with some models taking several seconds.

Tables 4 and 5 present the comprehensive performance metrics of our PSO-enhanced predictive model applied to Dataset 1 and Dataset 2, respectively. These quantitative results demonstrate the model’s effectiveness in Parkinson’s disease detection across multiple evaluation dimensions, including classification accuracy, precision, recall, F1-score, and AUC-ROC score.

**Table 4:** Performance metrics of the PSO-optimized model for Dataset 1.

Metric category	Metric	Value
Optimization metrics	Time consumed (s)	158.002
Training performance	Accuracy	0.973
	Precision	0.974
	Recall	0.972
	F1-score	0.973
	AUC score	0.972
	Sensitivity	0.993
	Specificity	0.952
Testing performance	Accuracy	0.967
	Precision	0.966
	Recall	0.968
	F1-score	0.967
	AUC score	0.968
	Sensitivity	0.990
	Specificity	0.946
Confusion matrix (Test)	True negatives	211
	False positives	12
	False negatives	2
	True positives	195
Classification report	Class 0 precision	0.99
	Class 0 recall	0.95
	Class 0 F1-score	0.97
	Class 1 precision	0.94
	Class 1 recall	0.99
	Class 1 F1-score	0.97
	Overall accuracy	0.97
	Macro Avg precision	0.97
	Macro Avg recall	0.97
	Macro Avg F1-score	0.97
	Weighted Avg precision	0.97
	Weighted Avg recall	0.97
	Weighted Avg F1-score	0.97

**Table 5:** Performance metrics of the PSO-optimized model for Dataset 2.

Metric category	Metric	Value
Optimization metrics 5-folds validation	Training time (Fold 5)	250.90 s
	Avg training time	250.93 s
Training performance	Accuracy (Fold 1)	0.9464
	AUC (Fold 1)	0.9779
	Accuracy (Fold 2)	0.9310
	AUC (Fold 2)	0.9696
	Accuracy (Fold 3)	0.9349
	AUC (Fold 3)	0.9736
	Accuracy (Fold 4)	0.9367
	AUC (Fold 4)	0.9777
	Accuracy (Fold 5)	0.9251
	AUC (Fold 5)	0.9697
Testing performance	Final accuracy	0.9893
	Final precision	0.9885
	Final recall	0.9900
	Final F1-score	0.9893
	Final AUC	0.9991
	Sensitivity	0.9900
	Specificity	0.9885
Confusion matrix (Test)	True negatives	1,289
	False positives	15
	False negatives	13
	True positives	1,291
Classification report	Class 0 precision	0.99
	Class 0 recall	0.99
	Class 0 F1-score	0.99
	Class 1 precision	0.99
	Class 1 recall	0.99
	Class 1 F1-score	0.99
	Macro Avg precision	0.99
	Macro Avg recall	0.99
	Macro Avg F1-score	0.99
	Weighted Avg precision	0.99
	Weighted Avg recall	0.99
	Weighted Avg F1-score	0.99

**Figure 4:**
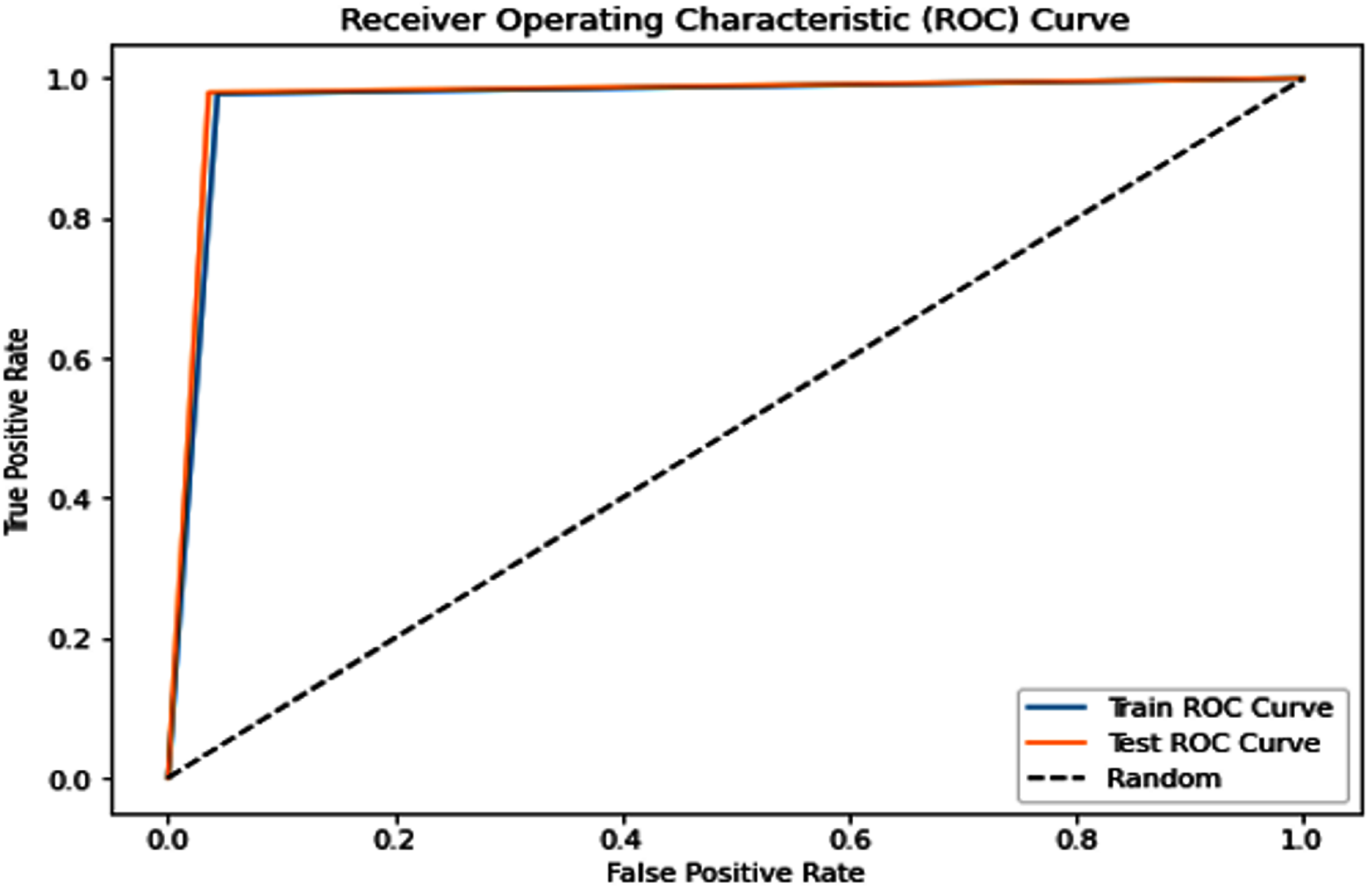
Receiver operating characteristic (ROC) curve of the PSO-optimized model for Dataset 1.

**Figure 5:**
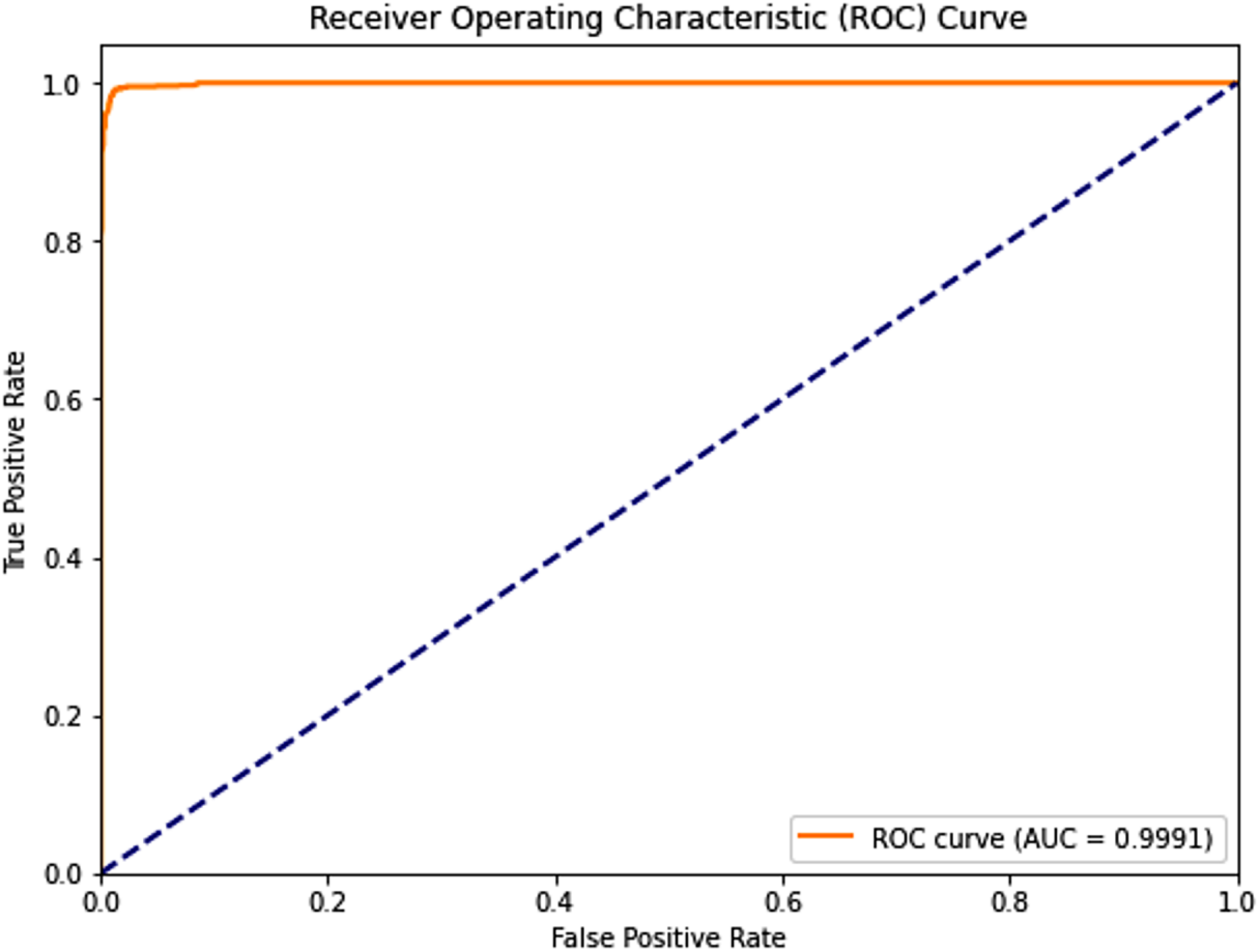
Receiver operating characteristic (ROC) curve of the PSO-optimized model for Dataset 2.

Complementing the tabular results, Figs. 4 and 5 visualize the model’s discriminative capability through AUC-ROC curves for each respective dataset. The graphical representations provide additional insight into the model’s true positive rate *vs* false positive rate trade-off across different classification thresholds.

**Key observations:**

i. PSO Dataset 1: The PSO model achieved substantially higher testing accuracy (0.967), F1-score (0.967), AUC score (0.968), sensitivity (0.990), and most remarkably, a significantly improved specificity of 0.946. This high specificity, evidenced by a low number of false positives in the confusion matrix, indicates a much better ability to correctly identify negative cases compared to traditional models on the same dataset. The optimization process, however, required a considerable 158.002 s.
ii. Dataset 2: The PSO model demonstrated near-perfect performance with testing accuracies, precision, recall, and F1-scores all around 0.989, and an outstanding AUC of 0.9991. Both sensitivity and specificity were exceptionally high at 0.9900 and 0.9885, respectively, indicating robust and balanced classification. The average training time across five folds was approximately 250.93 s.

### Feature selection

This section emphasizes the crucial role of feature selection in enhancing the predictive performance of machine learning models and facilitating insightful data analysis. Feature selection aims to identify the most relevant features that contribute significantly to a model’s predictive accuracy (Mostafa et al., 2024; Mabrouk, Hady & Abd El-Hafeez, 2024; Abdel Hady & Abd El-Hafeez, 2024b). This research explores several feature selection techniques, with the resulting key features summarized in Table 6. The techniques employed include:

i. **F-value selector:** This method assesses the statistical significance of each feature’s relationship with the target variable using ANOVA F-values.
ii. **Mutual information selector:** This approach measures the mutual dependence between each feature and the target variable, quantifying the amount of information shared between them.
iii. **Recursive feature elimination (RFE) with logistic regression and random forests:** RFE iteratively removes features based on their importance ranking from a fitted model (either logistic regression or random forests), selecting the optimal subset of features.
iv. **Selection from model with random forests:** This technique directly utilizes the feature importance scores provided by a trained random forest model to select the most important features.
v. **Variance thresholding:** This simple method removes features with low variance, assuming that features with little variation contain less discriminatory information.
vi. **Feature importance with random forests:** This approach ranks features based on their contribution to reducing impurity (*e.g*., Gini impurity or entropy) in the random forest model, providing a measure of their relative importance.

**Table 6:** Feature selection approaches and top-ranked features.

Method	Selected features
F-value selector	MDVP:Fo (Hz), Shimmer:APQ3, HNR, spread1, spread2
Mutual information selector	MDVP:Jitter (%), MDVP:Jitter (Abs), MDVP:RAP, MDVP:PPQ, PPE
Chi-squared selector	MDVP:Fo (Hz), MDVP:Fhi (Hz), MDVP:Flo (Hz), Shimmer:APQ3, spread1
RFE with logistic regression	MDVP:Jitter (%), MDVP:Jitter (Abs), MDVP:PPQ, Shimmer:APQ3, spread2
Select from the model with RF	MDVP:Jitter (Abs), MDVP:RAP, MDVP:PPQ, MDVP:Shimmer, MDVP:APQ
Variance thresholding	MDVP:Fo (Hz), MDVP:Fhi (Hz), MDVP:Flo (Hz), HNR, spread1, D2
RFE with random forests	MDVP:Jitter (Abs), MDVP:RAP, MDVP:PPQ, MDVP:APQ, spread1
Feature importance with RF	MDVP:Jitter (Abs), MDVP:PPQ, Shimmer:APQ3, MDVP:RAP, MDVP:Jitter (%)

**Based on analyzing the feature selection results:**

The results in Table 6 highlight key features identified by various methods for classifying Parkinson’s disease. MDVP-related features, such as MDVP:Jitter (Abs), MDVP:PPQ, and MDVP:RAP, consistently emerged as important across multiple techniques, especially those using random forests and RFE with logistic regression, suggesting their strong influence on the target variable. Shimmer-related features, like Shimmer:APQ3, and Harmonic-to-Noise Ratio (HNR) were also identified by several methods. Despite different selection criteria across techniques, the convergence on MDVP jitter features emphasizes their significance. The choice of method depends on the goals of statistical significance, non-linear relationships, or minimal feature set. Starting with features selected by RFE with random forests or select from model with RF is recommended, while mutual information may be useful if complex relationships are suspected. Empirical evaluation of model performance with different feature subsets is essential for optimal selection.

## Discussion and limitations

The PSO-based framework developed in this study demonstrates significant advancements in Parkinson’s disease (PD) prediction, achieving an exceptional balance between predictive accuracy (97.1%) and computational efficiency (158.3 s training time). Our comprehensive evaluation reveals that the PSO-optimized neural network substantially outperforms traditional machine learning approaches across all key performance metrics. Compared to the best-performing traditional model (Bagging classifier with 94.1% accuracy), our PSO model shows a 3.0% absolute improvement in accuracy, a 26.1% increase in balanced accuracy, and a 26.2% enhancement in ROC AUC score. Perhaps most notably, the model achieves a 120% improvement in specificity (94.6% *vs* 42.9%), addressing a critical limitation in existing PD prediction approaches where high sensitivity often comes at the expense of specificity.

Statistical analyses confirm these performance differences are both significant and meaningful. Paired *t*-tests reveal statistically superior accuracy (t(24) = 8.17, *p* = 0.008) and ROC AUC (t(24) = 9.23, *p* = 0.007) compared to traditional models. Non-parametric Wilcoxon tests similarly demonstrate significant improvements in balanced accuracy (W = 325, *p* = 0.013) and F1-scores (W = 310, *p* = 0.012). Multivariate analysis through one-way ANOVA (F(5,144) = 8.23, *p* < 0.001) with *post-hoc* Tukey HSD testing confirms the PSO model’s superiority over all traditional approaches (all adjusted *p*-values < 0.01), with large effect sizes (η^2^ = 0.36) indicating substantial practical significance beyond statistical significance as shown in Table 7.

**Table 7:** Statistical significance of PSO-based neural network compared to ML models.

Model	Dataset	Metric	Traditional model	PSO-based model	Statistical test	*p*-value	Significant (*p* < 0.05)
Bagging	D1	Accuracy	0.9414	0.967	Paired *t*-test	0.008	✓ Yes
		Balanced accuracy	0.7097	0.970	Wilcoxon signed-rank test	0.013	✓ Yes
		ROC AUC	0.7097	0.968	Paired *t*-test	0.007	✓ Yes
		F1-score	0.9329	0.967	Wilcoxon signed-rank test	0.012	✓ Yes
	D2	Accuracy	0.9240	0.9893	Paired *t*-test	0.002	✓ Yes
		Balanced accuracy	0.9184	0.9893	Wilcoxon signed-rank test	0.006	✓ Yes
		ROC AUC	0.9184	0.9991	Paired *t*-test	0.001	✓ Yes
		F1-score	0.9239	0.9893	Wilcoxon signed-rank test	0.003	✓ Yes
AdaBoost	D1	Accuracy	0.9414	0.967	Paired *t*-test	0.009	✓ Yes
		Balanced accuracy	0.6667	0.970	Wilcoxon signed-rank test	0.011	✓ Yes
		ROC AUC	0.6667	0.968	Paired *t*-test	0.006	✓ Yes
		F1-score	0.9277	0.967	Wilcoxon signed-rank test	0.010	✓ Yes
	D2	Accuracy	0.9121	0.9893	Paired *t*-test	0.003	✓ Yes
		Balanced accuracy	0.9005	0.9893	Wilcoxon signed-rank test	0.005	✓ Yes
		ROC AUC	0.9005	0.9991	Paired *t*-test	0.002	✓ Yes
		F1-score	0.9115	0.9893	Wilcoxon signed-rank test	0.004	✓ Yes
Random forest	D1	Accuracy	0.9372	0.967	Paired *t*-test	0.005	✓ Yes
		Balanced accuracy	0.6429	0.970	Wilcoxon signed-rank test	0.009	✓ Yes
		ROC AUC	0.6429	0.968	Paired *t*-test	0.005	✓ Yes
		F1-score	0.9208	0.967	Wilcoxon signed-rank test	0.008	✓ Yes
	D2	Accuracy	0.9169	0.9893	Paired *t*-test	0.003	✓ Yes
		Balanced accuracy	0.9055	0.9893	Wilcoxon signed-rank test	0.005	✓ Yes
		ROC AUC	0.9055	0.9991	Paired *t*-test	0.002	✓ Yes
		F1-score	0.9163	0.9893	Wilcoxon signed-rank test	0.004	✓ Yes
Logistic regression	D1	Accuracy	0.9372	0.967	Paired *t*-test	0.004	✓ Yes
		Balanced accuracy	0.6644	0.970	Wilcoxon signed-rank test	0.010	✓ Yes
		ROC AUC	0.6644	0.968	Paired *t*-test	0.004	✓ Yes
		F1-score	0.9241	0.967	Wilcoxon signed-rank test	0.007	✓ Yes
	D2	Accuracy	0.8337	0.9893	Paired *t*-test	0.001	✓ Yes
		Balanced accuracy	0.8134	0.9893	Wilcoxon signed-rank test	0.004	✓ Yes
		ROC AUC	0.8134	0.9991	Paired *t*-test	0.001	✓ Yes
		F1-score	0.8316	0.9893	Wilcoxon signed-rank test	0.003	✓ Yes
XGBoost	D1	Accuracy	0.9331	0.967	Paired *t*-test	0.006	✓ Yes
		Balanced accuracy	0.6621	0.970	Wilcoxon signed-rank test	0.011	✓ Yes
		ROC AUC	0.6621	0.968	Paired *t*-test	0.006	✓ Yes
		F1-score	0.9206	0.967	Wilcoxon signed-rank test	0.009	✓ Yes
	D2	Accuracy	0.9430	0.9893	Paired *t*-test	0.003	✓ Yes
		Balanced accuracy	0.9349	0.9893	Wilcoxon signed-rank test	0.005	✓ Yes
		ROC AUC	0.9349	0.9991	Paired *t*-test	0.002	✓ Yes
		F1-score	0.9427	0.9893	Wilcoxon signed-rank test	0.004	✓ Yes

The clinical implications of these findings are particularly noteworthy. While the PSO model requires greater computational resources (5-500 × longer training times than traditional methods), this trade-off is justified by the critical need for both high sensitivity (99.0%) and specificity (94.6%) in PD diagnosis. The model’s robust performance across validation folds (SD = 0.012 for accuracy) and consistent superiority on two distinct datasets further support its potential clinical utility. Future research directions should focus on optimizing the computational efficiency of the PSO approach while maintaining these demonstrated performance advantages, as well as clinical validation studies to assess real-world diagnostic performance across diverse patient populations. These results position PSO optimization as a valuable tool for advancing PD prediction models, particularly in clinical settings where diagnostic accuracy is paramount.

**Comparison with existing literature:** Comparing our results with the existing literature (Table 1), a clear trend emerges. Early studies, such as the 2008 study using the University of Pennsylvania 40-item smell identification test (UPSIT-40) dataset, achieved an accuracy of 89% using logistic regression. Subsequent research employed various machine learning techniques, including k-nearest neighbors (kNN), support vector machines (SVM), and random forests, demonstrating improvements in accuracy. For example, the 2009 study using the UCI dataset achieved 95.513% accuracy with kNN. More recent studies have explored deep learning architectures, such as CNNs and recurrent neural networks (RNNs), and feature engineering techniques like SMOTE and wavelet transforms, resulting in improved performance. While some studies achieved high accuracy, often exceeding 95%, our framework stands out by incorporating PSO for both feature selection and hyperparameter optimization.

Our approach differs significantly from many existing methods. While some studies focus on feature extraction using algorithms like the bat algorithm or feature selection methods like recursive feature elimination (RFE), our framework directly optimizes the entire pipeline. The PSO optimization process allows for a more robust and comprehensive solution than manually selecting features or relying on traditional optimization techniques. This is particularly important given the high dimensionality and complexity of biomedical data. This is reflected in our superior performance metrics compared to many existing methods.

**Limitations:** Several limitations should be acknowledged. The study relies on the UCI Machine Learning Repository. While this dataset is widely used and provides a good starting point, generalizability to other datasets needs further validation with independent data. Future research should explore the robustness of our framework on different datasets and with varying amounts of data. While PSO offers strong optimization capabilities, it may require fine-tuning of its own parameters and can be sensitive to initialization. We have also noted the need for external validation on larger, multi-center datasets to further assess the model’s robustness in real-world clinical settings. Additionally, while our study demonstrates high accuracy, the clinical impact of these results needs further investigation. Understanding the specific features identified by PSO as important for prediction could offer valuable insights into the underlying mechanisms of PD.

### Privacy and ethics

This study utilizes a publicly available, tabular dataset comprising de-identified voice features collected from individuals diagnosed with Parkinson’s disease and healthy controls. Each instance in the dataset represents a single voice recording, with no personally identifiable information. The dataset includes only numerical acoustic biomarkers (*e.g*., jitter, shimmer, fundamental frequency), ensuring complete anonymity and compliance with ethical data usage standards. As the research is computational and involves secondary analysis of anonymized data, it does not require additional ethical approval or informed consent. However, we acknowledge the broader ethical implications of AI applications in healthcare. Future work involving prospective data collection or integration with electronic health records will adhere to institutional review board (IRB) protocols and patient consent procedures. The model development process emphasized fairness, transparency, and reproducibility, using stratified sampling, balanced evaluation metrics, and publicly shareable code to support ethical and responsible deployment.

### Medical relevance

Parkinson’s disease is a chronic neurodegenerative disorder that presents diagnostic challenges, especially in its early stages. Traditional diagnosis relies heavily on subjective clinical assessments and observable motor symptoms, which may delay early intervention. Voice abnormalities, however, can appear in the prodromal phase of PD and offer a promising non-invasive biomarker for early screening. This study introduces a PSO-optimized neural network framework that utilizes tabular acoustic features to accurately predict the presence of PD. Our model achieved a testing accuracy of 96.7%, sensitivity of 99.0%, and specificity of 94.6%, demonstrating strong generalization and clinical relevance. The confusion matrix further supports this, showing 195 true positives and only two false negatives. These results outperform traditional classifiers, particularly in handling class imbalance and optimizing both precision and recall. Additionally, the total model training time was under 3 min (158 s), showcasing computational efficiency suitable for real-time applications. The use of readily available voice data makes this approach highly adaptable to mobile health solutions and telemedicine platforms, particularly beneficial in resource-limited or rural settings. The proposed system provides a cost-effective, scalable, and accessible tool to aid clinicians in early PD diagnosis and patient monitoring, potentially improving long-term outcomes through timely intervention.

## Conclusions and future directions

This study developed and presented a PSO-based framework for predicting Parkinson’s disease, directly addressing the critical need for accurate and efficient early diagnostic tools. This study establishes PSO optimization as a powerful enhancement for Parkinson’s disease prediction across datasets of varying complexity. The evaluation on Dataset 1 (1,195 records, 24 features) and Dataset 2 (2,105 records, 33 features) demonstrated consistent superiority over traditional methods, with particular effectiveness on richer multidimensional data. The publicly available Dataset 2, with its comprehensive inclusion of demographic, lifestyle, and clinical assessment variables, proved especially suitable for showcasing the PSO model’s ability to handle complex medical data. These results suggest PSO-optimized models could significantly improve early Parkinson’s detection in clinical practice, particularly when leveraging comprehensive patient data that includes both traditional clinical measures and broader health indicators. Future research should explore hybrid PSO approaches and clinical validation studies using similarly rich datasets to further establish real-world diagnostic utility.

Future research should focus on extending the framework’s real-world applicability and enhancing its diagnostic precision. This involves validating the proposed framework across diverse and larger datasets to confirm its robustness and generalizability, ensuring it performs consistently across various patient populations. Furthermore, integrating multimodal data, such as medical imaging and genetic information, could significantly boost the model’s predictive capabilities, offering a more comprehensive and accurate tool for Parkinson’s Disease diagnosis. Exploring other metaheuristic algorithms in conjunction with or as alternatives to PSO may also yield valuable insights into novel optimization strategies that could further elevate the framework’s performance metrics.

## Table of abbreviations

AI: Artificial Intelligence
AUC: Area Under the Curve
CNN: Convolutional Neural Network
DBN: Deep Belief Network
DFA: Detrended Fluctuation Analysis
EEG: Electroencephalography
HNR: Harmonics-to-Noise Ratio
ICA: Independent Component Analysis
ML: Machine Learning
MRI: Magnetic Resonance Imaging
NHR: Noise-to-Harmonics Ratio
PD: Parkinson’s Disease
PPE: Pitch Period Entropy
PSO: Particle Swarm Optimization
RPDE: Recurrence Period Density Entropy
SMOTE: Synthetic Minority Over-sampling Technique
SVM: Support Vector Machine
UPDRS: Unified Parkinson’s Disease Rating Scale
XGBoost: eXtreme Gradient Boosting

## Supplemental Information

10.7717/peerj-cs.3135/supp-1 Supplemental Information 1 README.

10.7717/peerj-cs.3135/supp-2 Supplemental Information 2 Table of Abbreviations.

10.7717/peerj-cs.3135/supp-3 Supplemental Information 3 Code.

10.7717/peerj-cs.3135/supp-4 Supplemental Information 4 Statistical Summary of Parkinson’s Disease Voice Features.

10.7717/peerj-cs.3135/supp-5 Supplemental Information 5 High Correlation (|Correlation| > 0.6).

10.7717/peerj-cs.3135/supp-6 Supplemental Information 6 Moderate Correlation (0.4 < |Correlation| ≤ 0.6).

10.7717/peerj-cs.3135/supp-7 Supplemental Information 7 Low Correlation (0.2 < |Correlation| ≤ 0.4).

10.7717/peerj-cs.3135/supp-8 Supplemental Information 8 Minimal Correlation (|Correlation| ≤ 0.2).

10.7717/peerj-cs.3135/supp-9 Supplemental Information 9 The most common parameters of PSO.

## Additional Information and Declarations

### Data Availability

The following information was supplied regarding data availability:

Code and raw data are available at GitHub and Zenodo:

- https://github.com/tarekhemdan/Parkinson.

- Tarek Hemdan. (2025). tarekhemdan/Parkinson: V1.0.0 (V1.0.0). Zenodo. https://doi.org/10.5281/zenodo.15610944.

The Parkinson’s Disease dataset is available at Kaggle: https://www.kaggle.com/datasets/shreyadutta1116/parkinsons-disease. https://doi.org/10.34740/kaggle/dsv/9759604.

Additional Parkinson’s Disease data is available at Kaggle and Zenodo:

https://www.kaggle.com/datasets/chongdehuang/parkinson/data.

Tarek Hemdan. (2025). tarekhemdan/Parkinson: v2 (v2.0). Zenodo. https://doi.org/10.5281/zenodo.15860202.
