# Supplementary material for: Particle swarm optimization framework for Parkinson’s disease prediction
Source: PeerJ Comput Sci. 2025 Sep 11;11:e3135. doi: 10.7717/peerj-cs.3135 (PMC12453757; doi:10.7717/peerj-cs.3135)
Supplement: Supplemental Information 2 [file peerj-cs-11-3135-s002.docx]

| **Abbreviation** | **Full Form** |
| --- | --- |
| AI | Artificial Intelligence |
| AUC | Area Under the Curve |
| CNN | Convolutional Neural Network |
| DBN | Deep Belief Network |
| DFA | Detrended Fluctuation Analysis |
| EEG | Electroencephalography |
| HNR | Harmonics-to-Noise Ratio |
| ICA | Independent Component Analysis |
| ML | Machine Learning |
| MRI | Magnetic Resonance Imaging |
| NHR | Noise-to-Harmonics Ratio |
| PD | Parkinson's Disease |
| PPE | Pitch Period Entropy |
| PSO | Particle Swarm Optimization |
| RPDE | Recurrence Period Density Entropy |
| SMOTE | Synthetic Minority Over-sampling Technique |
| SVM | Support Vector Machine |
| UPDRS | Unified Parkinson's Disease Rating Scale |
| XGBoost | eXtreme Gradient Boosting |
