# Supplementary material for: Particle swarm optimization framework for Parkinson’s disease prediction
Source: PeerJ Comput Sci. 2025 Sep 11;11:e3135. doi: 10.7717/peerj-cs.3135 (PMC12453757; doi:10.7717/peerj-cs.3135)
Supplement: Supplemental Information 4 [file peerj-cs-11-3135-s004.docx]

| **Statistic** | **MDVP:Fo**  **(Hz)** | **MDVP:Fhi**  **(Hz)** | **MDVP: Flo**  **(Hz)** | **MDVP: Jitter**  **(%)** | **MDVP: Jitter**  **(Abs)** | **MDVP:RAP** | **MDVP:**  **PPQ** | **Jitter:**  **DDP** | **MDVP: Shimmer** | **MDVP: Shimmer**  **(dB)** |
| --- | --- | --- | --- | --- | --- | --- | --- | --- | --- | --- |
| count | 195.0 | 195.0 | 195.0 | 195.0 | 195.0 | 195.0 | 195.0 | 195.0 | 195.0 | 195.0 |
| mean | 154.2 | 197.1 | 116.3 | 0.0 | 0.0 | 0.0 | 0.0 | 0.0 | 0.0 | 0.3 |
| std | 41.4 | 91.5 | 43.5 | 0.0 | 0.0 | 0.0 | 0.0 | 0.0 | 0.0 | 0.2 |
| min | 88.3 | 102.1 | 65.5 | 0.0 | 0.0 | 0.0 | 0.0 | 0.0 | 0.0 | 0.1 |
| 25% | 117.6 | 134.9 | 84.3 | 0.0 | 0.0 | 0.0 | 0.0 | 0.0 | 0.0 | 0.1 |
| 50% | 148.8 | 175.8 | 104.3 | 0.0 | 0.0 | 0.0 | 0.0 | 0.0 | 0.0 | 0.2 |
| 75% | 182.8 | 224.2 | 140.0 | 0.0 | 0.0 | 0.0 | 0.0 | 0.0 | 0.0 | 0.4 |
| max | 260.1 | 592.0 | 239.2 | 0.0 | 0.0 | 0.0 | 0.0 | 0.1 | 0.1 | 1.3 |
| **Table 3 (b): Statistical Summary (Columns 11–21)** | | | | | | | | | | |
| **Statistic** | **Shimmer:**  **DDA** | **NHR** | **HNR** | **status** | **RPDE** | **DFA** | **spread1** | **spread2** | **D2** | **PPE** |
| count | 195.00 | 195.00 | 195.00 | 195.00 | 195.00 | 195.00 | 195.00 | 195.00 | 195.00 | 195.00 |
| mean | 0.05 | 0.02 | 21.89 | 0.75 | 0.50 | 0.72 | -5.68 | 0.23 | 2.38 | 0.21 |
| std | 0.03 | 0.04 | 4.43 | 0.43 | 0.10 | 0.06 | 1.09 | 0.08 | 0.38 | 0.09 |
| min | 0.01 | 0.00 | 8.44 | 0.00 | 0.26 | 0.57 | -7.96 | 0.01 | 1.42 | 0.04 |
| 25% | 0.02 | 0.01 | 19.20 | 1.00 | 0.42 | 0.67 | -6.45 | 0.17 | 2.10 | 0.14 |
| 50% | 0.04 | 0.01 | 22.09 | 1.00 | 0.50 | 0.72 | -5.72 | 0.22 | 2.36 | 0.19 |
| 75% | 0.06 | 0.03 | 25.08 | 1.00 | 0.59 | 0.76 | -5.05 | 0.28 | 2.64 | 0.25 |
| max | 0.17 | 0.31 | 33.05 | 1.00 | 0.69 | 0.83 | -2.43 | 0.45 | 3.67 | 0.53 |
