# Supplementary material for: Particle swarm optimization framework for Parkinson’s disease prediction
Source: PeerJ Comput Sci. 2025 Sep 11;11:e3135. doi: 10.7717/peerj-cs.3135 (PMC12453757; doi:10.7717/peerj-cs.3135)
Supplement: Supplemental Information 5 [file peerj-cs-11-3135-s005.docx]

| Rank | Correlation | Feature 1 | Feature 2 | Clinical Recommendation |
| --- | --- | --- | --- | --- |
| 1 | +0.831 | PPE | spread1 | **High priority**: Closely monitor both features as they show a strong positive relationship, indicating that changes in one may directly affect the other. |
| 2 | -0.801 | HNR | MDVP:Shimmer (dB) | **Critical concern**: The strong negative correlation suggests that as the shimmer increases, the harmonic-to-noise ratio decreases. Evaluate voice quality and consider interventions. |
| 3 | -0.669 | HNR | spread1 | **Monitor closely**: Significant negative relationship; may indicate worsening voice quality requiring potential therapy. |
| 4 | +0.631 | MDVP:Shimmer (dB) | spread1 | **Track changes**: Positive correlation indicates that monitoring both features can provide insights into patient voice quality. |
| 5 | +0.593 | MDVP:Flo (Hz) | MDVP:Fo (Hz) | **Evaluate frequency changes**: Clinically assess pitch variations to better understand impacts on speech. |
| 6 | -0.593 | D2 | HNR | **Investigate further**: The significant negative correlation may indicate potential voice disorders; consider speech therapy. |
