# Supplementary material for: Particle swarm optimization framework for Parkinson’s disease prediction
Source: PeerJ Comput Sci. 2025 Sep 11;11:e3135. doi: 10.7717/peerj-cs.3135 (PMC12453757; doi:10.7717/peerj-cs.3135)
Supplement: Supplemental Information 6 [file peerj-cs-11-3135-s006.docx]

| Rank | Correlation | Feature 1 | Feature 2 | Clinical Recommendation |
| --- | --- | --- | --- | --- |
| 7 | -0.587 | HNR | PPE | **Consider monitoring**: Negative correlation suggests voice quality may impact predictability; adjust treatment as necessary. |
| 8 | +0.578 | MDVP:Shimmer (dB) | PPE | **Enhance monitoring**: Positive correlation indicates that tracking both features can provide insights into patient progress. |
| 9 | +0.547 | spread1 | spread2 | **Regular assessments**: Monitor both features for understanding therapy outcomes. |
| 10 | -0.514 | HNR | RPDE | **Further investigation**: A negative correlation suggests that voice quality may influence complexity measures; assess for treatment adjustments. |
| 11 | +0.498 | RPDE | spread1 | **Monitor regularly**: Positive correlation implies that both features should be tracked to evaluate speech characteristics. |
| 12 | +0.487 | PPE | spread2 | **Track for changes**: Regular evaluations can inform treatment efficacy. |
