# Supplementary material for: Particle swarm optimization framework for Parkinson’s disease prediction
Source: PeerJ Comput Sci. 2025 Sep 11;11:e3135. doi: 10.7717/peerj-cs.3135 (PMC12453757; doi:10.7717/peerj-cs.3135)
Supplement: Supplemental Information 7 [file peerj-cs-11-3135-s007.docx]

| Rank | Correlation | Feature 1 | Feature 2 | Clinical Recommendation |
| --- | --- | --- | --- | --- |
| 13 | +0.484 | D2 | spread1 | **Routine evaluations**: Monitor these features to maintain awareness of voice characteristics. |
| 14 | +0.483 | MDVP:Shimmer (dB) | Shimmer:DDA | **Consider tracking**: May provide insights into amplitude stability; monitor for changes. |
| 15 | +0.480 | D2 | MDVP:Shimmer (dB) | **Regular monitoring**: Track changes to assess treatment impact. |
