# Supplementary material for: Particle swarm optimization framework for Parkinson’s disease prediction
Source: PeerJ Comput Sci. 2025 Sep 11;11:e3135. doi: 10.7717/peerj-cs.3135 (PMC12453757; doi:10.7717/peerj-cs.3135)
Supplement: Supplemental Information 8 [file peerj-cs-11-3135-s008.docx]

| Rank | Correlation | Feature 1 | Feature 2 | Clinical Recommendation |
| --- | --- | --- | --- | --- |
| 99 | -0.084 | MDVP:Fo (Hz) | MDVP:RAP | **Maintain routine monitoring**: Minimal correlation; keep in mind for overall assessments. |
| 100 | -0.083 | MDVP:Fo (Hz) | MDVP:Shimmer (dB) | **General observation**: Track as part of routine checks; no immediate action needed. |
