# Supplementary material for: Particle swarm optimization framework for Parkinson’s disease prediction
Source: PeerJ Comput Sci. 2025 Sep 11;11:e3135. doi: 10.7717/peerj-cs.3135 (PMC12453757; doi:10.7717/peerj-cs.3135)
Supplement: Supplemental Information 9 [file peerj-cs-11-3135-s009.docx]

| **Parameter** | **Symbol** | **Parameter Value** |
| --- | --- | --- |
| **No. of particles** | *P_size_* | ***P_size_∈ [10…40] Particles*** |
| **Maximum velocity** | *V_max_* | ***V_max_ = 0.2*** |
| **Minimum velocity** | *V_min_* | ***V_min_= - V_max_*** |
| **Inertia weight** | *w* | ***w= ((T_max_ - G) * (0.9 - 0.4) / T_max_) + 0.4*** |
| **First acceleration parameter** | *c_1_* | ***c_1_∈ [0.5,2]*** |
| **Second acceleration parameter** | *c_2_* | ***c_2_=c_1_  or c_1_+c_2_≤ 4*** |
| **Diversity of the population maintenance** | *r_1_,r_2_* | ***r_1_,r_2_∈ [0,1]*** |
| **Iteration** | ***T_max_*** | ***T_max_ ≤ 30000*** |
